# Commentary on 2022 guidelines on clinical trial design in cluster headache and further suggestions

**DOI:** 10.1186/s10194-024-01732-3

**Published:** 2024-03-07

**Authors:** Ioana Medrea, Stewart J. Tepper, Donliang Wang, Paul G. Mathew, Mark Burish

**Affiliations:** 1 SUNY Upstate Medical University, Syracuse, New York, USA; 2 The New England Institute for Neurology and Headache, 30 Buxton Farms Rd, Stamford, CT USA; 3 Harvard Medical School, Boston, MA USA; 4 Mass General Brigham Health, Department of Neurology, Foxboro, MA USA; 5 Atrius Health, Department of Neurology, Quincy, MA USA; 6 UTHealth Houston, Department of Neurosurgery, Houston, TX USA

**Keywords:** Cluster headache trial, Methodology cluster headache design, Future directions cluster headache trials

## Abstract

**Background:**

New guidelines for cluster headache clinical trials were recently published. We welcome these new guidelines and raise additional considerations in trial methodologies.

**Main body:**

We present non-inferiority trials to overcome ethical issues with placebo use, and additionally discuss issues with trial recruitment.

**Conclusions:**

We highlight some possible issues and solutions to be considered with the recently published cluster headache trial guidelines.

## Background

Cluster headache is the most common trigeminal autonomic cephalalgia (TAC) with a prevalence of 1 in 1000 individuals [1, 2]. Cluster headache involves a unilateral, usually retro-orbital, severe headache with ipsilateral autonomic features and/or agitation. Differentiating cluster headache from other TACs is the duration of each attack, which can last 15 minutes to 3 hours with up to 8 attacks per day, and the treatment response (in particular cluster is usually not responsive to indomethacin unlike other TACs it may overlap). It is considered to be one of the most painful conditions known to affect humans [3]. The International Headache Society (IHS) released guidelines for treatment trials in cluster headache in December 2022 [4]. This is a comprehensive and much needed resource to ensure rigor in cluster headache trials.

We applaud this new resource, as an update for cluster treatment trial guidelines is overdue considering the new treatment modalities such as anti-calcitonin gene related peptide (CGRP) monoclonal antibodies (mAbs) and neuromodulation and the evolution in clinical trial design since the last guidelines iteration [5]. Moreover, there is need for further clarification of best practices in cluster headache clinical trials given the recent successes and failures of several trials. Guidelines by nature are aspirational, but to exemplify best practice in trial design, some issues are worth highlighting particular to cluster headache that have led to limited recruitment in trials, lack of adequate sample size, and issues with trial completion [6–8]. While we agree with most of the new trial recommendations, there are 3 recommendations that we would like to discuss further especially as these pertain to episodic cluster headache trial design.

### The use of placebo versus active comparators

Placebo-controlled trials have been the recommended standard for cluster headache acute and preventive trials. However, this may lead to poor enrollment, especially in preventive trials, given the excruciating pain of cluster headache and the associated reduced likelihood of patients willing to risk being assigned to the placebo arm of a study [6, 9]. Placebo-controlled designs are recommended by the new guidelines. However, it is mentioned that active comparators can be used when both therapies have proven effective over placebo [4]. We would like to emphasize, and strongly encourage, the use of active comparators as a potential way to increase enrollment. There are well established methods for non-inferiority, equivalence, and superiority testing that are accepted in other fields of medicine [10]. When an effective treatment already exists, it may be appropriate and adequate to know that a new treatment is as good as the old treatment [11]. The new treatment may be more accessible, safer, or may be used in patients with failure of the first treatment. When there is a standard of care in prevention for such a painful condition, placebo arms have inadvertently, but historically, reduced enrollment.

For active comparator trials, consideration should be given to utilizing:

- Superiority trials to demonstrate one treatment has greater efficacy than another.
- Equivalence trials to demonstrate one treatment is as effective as another.
- Non-inferiority trials to demonstrate that one treatment is not less effective than another [12].

There are, of course, some issues that need to be addressed prior to the use of these trial designs in cluster headache.

There is a question of appropriateness of use of these designs given the possibility of a placebo effect. In acute trials it is well established that the placebo effect exists and is quite variable [13]. In the proposed active comparator trial designs, the variable placebo effect seems to be related to the route of administration, and this will be discussed below (see Table 1). In preventive trials, a large part of the presumed placebo effect is likely not a placebo effect but rather the natural remission of a given cluster headache period or cycle. In those trials that enrolled patients only at the onset of a cluster period, the placebo effect was non-existent or very low [14–16], while in other trials it is noted that “placebo effect” resolution of cluster headaches is observed more often as the study carries on [7, 17]. We agree that when there is a variable and possibly large placebo analgesic effect, there should a placebo comparator [10], but we believe that the purported placebo effect in episodic cluster headache is neither variable nor large when preventive studies of cluster headache start at the onset of a headache period. The historical placebo preventive effects in terms of headache reduction are relatively predictable (see Table 2). When looking at trials enrolling early at the onset of a cycle this effect is questionable, as seen in studies by Leone and colleagues published in 1996 and 2000. When enrolling later in the cluster period, the effect on cluster attack reduction is in the range of 4–6 less headaches per week.

**Table 1:** Relevant studies of acute treatment headache response at 15 or 30 min

Trial and Year	Placebo	Suma SC	Zolmi NS	O_**2**_ low	O_**2**_ high	Suma NS	Headache Response Timeline Analyzed
**Ekbom 1991** [21]	25%, *n* = 39	74%, n = 39					15 min
**Ekbom 1993** [20]	35%, *n* = 88	75%, *n* = 93					15 min
**Cittadini 2006** [24]	23%, *n* = 61		42%, *n* = 65				30 min
**Rapoport 2007** [22]	30%, *n* = 59		50%, *n* = 52				30 min
**Fogan 1985** [27]	33%, *n* = 63			68%, *n* = 74			15 min
**Cohen 2009** [26]	20%, *n* = 148				78%, *n* = 150		15 min
**Dirkx 2018** [9]				14%, *n* = 14	33%, *n* = 15		15 min
**Van Viet 2003** [23]	26%, *n* = 77					57%, *n* = 77	30 min

Used with permission from Medrea et al., Headache 2022 [19]

*O*_2_ oxygen, *SC* subcutaneous, *nVNS* non-invasive vagal nerve stimulation, *NS* nasal spray, *Suma* sumatriptan; Zolmi, zolmitriptan

**Table 2:** Preventive trials that feature changes in headache frequency from baseline to 2-weeks, ≥50% responder rates, and adverse events

Trial and year	Treatment	ECH	Baseline frequency active Rx per week ± SD	Rx reduction to week 2 ± SD	Baseline frequency placebo per week ± SD	Placebo reduction to week 2 ± SD	Active Treatment 50% response	Placebo 50% resp
**Goadsby 2019** [30]	Galcanezumab	All	17.3 ± −10	−8.8 ± 12.1, *n* = 57	17.8+/− 10.1	− 4.5 ± 10.5, *n* = 49	39%, *n* = 57	25%, *n* = 49
**Leone 1996** [16]	Melatonin	90%	23.1	− 12.7 ± 16.0, *n* = 10	16.73	0.8 ± 11.4, *n* = 10	NA	NA
**Leone 2000** [14]	Verapamil	All	13.4	−9.2, *n* = 15	9.59	1.7, *n* = 15	80%, *n* = 15	0%, *n* = 15
**Saper 2002** [31]	Intranasal civamide	ECH	12.5	−8.4 ± 9.1, *n* = 18	10.8	−3.6 ± 8.7 *n* = 10	NA	NA
**Tronvik 2013** [7]	Candesartan	ECH	14.3 ± 9.2	−8.7, *n* = 19	16.8	−6.2, *n* = 13	63%, *n* = 19	46%, n = 13

Used with permission from Medrea et al., Headache 2022 [19]

*AE* adverse events, *ECH* episodic cluster headache, *NA* not available, *PLAC* placebo, *Rx* prescription drug

In situations where active comparators are appropriate, margins need to be a priori defined [10]. The issues with active comparator trials can be overcome if non-inferiority margins are based on previous historical data, additionally effect sizes can be estimated for the control drug, and lastly sample size is calculated prior to the commencement of the trial [18]. To ensure consistent active comparator responses, we propose the following recommendations for non-inferiority active comparator trials:

- Include similar populations to the original trials in question.
- Begin treatment in the first 1–2 weeks of a cluster headache period for preventive trials and collect outcomes early in the cluster headache episode to limit chances of natural remission. Enrollment can occur in between cycles.
- Compare active comparator outcomes to previous expected values to validate.

**Acute Treatments - Proposal for Non-inferiority Margins** We propose these margins vary by administration type:

- Subcutaneous injections: 20% non-inferiority margin
- Nasal sprays: 10% non-inferiority margin
- Inhalations (i.e. oxygen): 20% non-inferiority margin

We base our conclusions on historical data highlighted in Table 1. The largest placebo response seen in terms of headache relief for cluster headache was 35% for the injectable sumatriptan formulation, and treatment response in this category was in the range of 75% [19–21]. A 20% non-inferiority margin may be reasonable for injectable formulations, if expected to perform as well as sumatriptan injectable based on the cited trials [25, 31] and the planned pilot trials. For nasal spray formulations, the largest placebo headache relief response was 30%, and the response rate varied between 42% and 57% for the various treatments [24, 26, 28, 32]. A 10% non-inferiority margin may be set for nasal spray formulations, if expected to perform as well as sumatriptan or zolmitriptan nasal spray based on pilot trials. For different oxygen delivery devices such as high flow non-rebreather mask versus demand valve oxygen [25], placebo rates peak at 33%, and treatment rates of relief reach 68% and 78% [19, 26, 27]. A 20% non-inferiority margin may be considered for oxygen delivery formulations, if expected to perform as well as non-rebreather oxygen based on pilot trials. We urge caution in the design of acute trials when using non-inferiority margins, as in acute trials the placebo response is robust and may be variable. These types of designs may be more reasonable when comparing classes of therapies that are already known to be effective rather than in new therapies.

### Preventive treatments - proposal for a non-inferiority trial design

As an example of a preventive cluster headache trial design using active comparators, we present our proposed conditions for a non-inferiority preventive trial. The active comparator, importantly, has proven efficacy from prior placebo-controlled cluster headache trials. We propose:

- Active comparator: either galcanezumab (episodic only), verapamil, and melatonin [28, 29] choosing the appropriate treatment to optimize blinding.
- Non-inferiority margin: a reduction in headache frequency of − 4 attacks per week of the new treatment over the active comparator.
- Sample size: adequate according to sample size calculations estimations using an effect of − 9.2 attacks/week or more, a non-inferiority margin of 2 or 4 as seen fit (see more below on guidance) [30].
- Comparison with previous placebo-controlled trials to ensure response is in line with historical data. Both the new treatment and the active comparator should show a reduction of at least − 9 attacks/week.
  We examined prior cluster headache clinical trials to arrive at this current proposal. Table 2 highlights some considerations for weekly attack frequency reduction. With active treatment, weekly attack reduction has varied between − 8.4 to − 12.7 attacks [7, 14, 16, 31–33], but has been in the range of − 8.4 to − 9.2 attacks per week in most of these trials [7, 14, 16, 31–33]. For these same trials, placebo rates have varied between an increase in frequency of + 0.8 and + 1.7 attacks per week when the patients were enrolled in the first week of a cycle [14, 16], to a reduction of- 3.6, − 4.5 and up to − 6.2 attacks per week when they were not enrolled exclusively early in a cluster cycle.
  After going through this data, we propose that a reasonable non-inferiority margin, a reduction in headache frequency in the range of − 4 attacks per week of the new treatment over the active comparator. This would guarantee separation from placebo in all historical trials except for the outlier discussed below [7], and less than 50% reduction in treatment effect where patients are enrolled early in a cluster period (under 2 weeks). Admittedly a smaller non-inferiority margin of − 2 attacks per week would guarantee separation in all historical trials. The more stringent non-inferiority margin is needed because of an outlier, the larger − 6.2 attack reduction seen in the placebo arm by Tronvik et al. in the trial published in 2013 [7]. Again, this high placebo rate could likely be avoided if only allowing enrollment in the first 2 weeks of a cluster period, as likely a large part of this attack reduction was due to natural remission [7]. We propose the more conservative margin of − 2 attacks/week differential could be useful in regulatory trials or where a benefit over an active comparator is being sought. Assuming a standard deviation of 9, significance level of 5%, and power 80% the sample size per group would be 251 patients [34]. The more permissive margin of − 4 attacks/week allows for a smaller sample size in the trials. Assuming a standard deviation of 9, the sample size per group would be 63 patients [34]. This more permissive margin should be used in trials where recruitment may be difficult, the treatment being investigated is already in use as a third line management of cluster headache, or case series or other data of effectiveness exists, needing confirmatory evidence for establishment of an evidence base.

Trial populations should be consistent with prior cluster headache clinical trials to ensure similar active comparator results.. We propose that both the new treatment and the active comparator should show a reduction in the range of − 9.2 attacks/week [35, 36] or more as established in the literature or in a pilot trial, if the response is less than this the efficacy of the treatment should be in question.

We do not propose non-inferiority margins for 50% responder rate reduction as there were a limited number of studies reporting this, and the historical data was not as dependable. There have been some trials where the comparator arm was verapamil and not placebo, or oxygen delivery etc. These studies were more pragmatic trial designs evaluating for superiority [17, 37] or non-inferiority to current treatments [38].

### Considerations for a baseline period

#### A 1–2 week prospective baseline period versus a 3 day retrospective baseline period

The new trial guidelines recommend a baseline period of at least 1 week in all acute treatment trials, at least 1 week in preventive trials for episodic cluster headache, and at least 2 weeks in preventive trials for chronic cluster headache. In addition, the baseline data should be collected prospectively in all cases. However, one well-designed trial eschewed this recommendation, opting instead for a 3 day retrospective baseline [17]. A 3 day retrospective baseline would minimize chances of cycle remission and also limit period without exposure to preventive medications. Additionally, if analysis is undertaken with the exact Mantel-Haenszel test of common Poisson relative risk over the whole treatment window, or survival analysis, the baseline period becomes less important, as these tests do not depend on changes from a baseline but rather the rate of change over time.

### Primary endpoints

#### Acute trials: headache freedom at 15 minutes versus headache freedom at 30 minutes

The recommended primary endpoint from the new trial guidelines is headache freedom at 15 minutes. This can be justified for two reasons: 1) not wanting cluster patients to be without acute therapy for longer than this duration if on placebo, and 2) to avoid the natural cessation of an attack, since the ICHD3 defined duration of a cluster headache attack is 15–180 minutes. However, using this very stringent primary outcome, sumatriptan injection, [25] zolmitriptan nasal spray 10 mg [22], and high flow oxygen would pass the bar [26], but not nasal spray zolmitriptan 5 mg [22] or the 20 mg sumatriptan nasal spray [23]. Thus the recommended outcome measure would have kept both zolmitriptan and sumatriptan nasal sprays from being recommended for clinical use, even though both American and European guidelines consider them efficacious [28, 39]. It seems reasonable to also allow efficacy evaluations at 30 minutes, whether headache relief, as utilized in many acute trials of approved medications, or freedom, especially as the timeline of onset of some nasal spray formulations is more likely to be 30 min. If the 30-minute primary endpoint is used, then studies should enroll patients whose attacks typically last at least 45 minutes. This is even more important for placebo studies than for active comparator studies. A 15-minute endpoint could still be used if it is preferred, especially if there is a desire to enroll all cluster headache patients and not just those with attacks that last at least 45 minutes. Thus, the 15-minute pain free outcome is aspirational and is sometimes met, but the severity of cluster headache and the dearth of treatments merits some flexibility on acceptable outcomes. An advantage of multiple acceptable outcome times is that for 15-minute pain relief, access to acute treatment becomes available, the standard is lowered for regulatory approval, and more therapies may be made accessible [32].

#### Preventive trials: weekly attack frequency versus different endpoints for placebo and active comparator studies

The recommended primary endpoint from the new trial guidelines is a change from baseline in the number of weekly attacks for the entire double-blind phase [3]. However, this can be limiting if the trial goes on for a longer period (over 4 weeks), as there is natural disease remission in both treatment and placebo groups, and it becomes difficult to show a difference between study groups at later time points [7]. In light of this consideration, we propose two alternative primary endpoints:

- Primary endpoint for trials with an active comparator. It may be necessary to have a small pilot to decide on the timeline of cessation of attacks with treatment, choose a comparison treatment with a similar timeline to cessation of headaches, and evaluate the number of headache attacks in that given week.
- Primary endpoint for placebo-controlled trials. In placebo-controlled trials, earlier cessation of headaches can be very important. A suggested analysis from a previous trial is the Mantel-Haenszel test of common Poisson relative risk over the whole treatment window [7], which would show separation from placebo with earlier cessation of attacks even if by the last trial period the groups are similar due to natural remission. There are some methods to address non-inferiority trial design using survival analysis and these can be further explored in future studies but were not explored for the current manuscript.

## Conclusions

In conclusion, we highlight some possible issues and solutions to be considered with placebo-controlled designs and primary endpoints as proposed in the recent clinical trial design guidelines for cluster headache. We present considerations for why placebo may not be necessary, especially in preventive trials of cluster headache, provided active treatment response is within the non-inferiority margins of historical control treatment responses and provided patients are within the first week of cluster cycle at enrollment to minimize the likelihood of cycle remission. Additionally, we propose some possible non-inferiority margins, which can vary depending on how stringent the trial design needs to be, and we propose that the more conservative measures may be considered for regulatory approval to limit exposure to placebo rather than prevention for many weeks. We propose some alternative statistical techniques that may be better suited for analysis of whole trial data, especially when taking into consideration the timeline of cessation of cluster attacks. We hope that these suggestions will be further tested in pilot trials and future clinical trials to prevent our patient population unnecessary exposure to placebo, emphasizing again that cluster headache is one of the most painful clinical conditions [3].

## Data Availability

No datasets were generated or analysed during the current study.
